# Food: Cooking and Catering

**Published:** 1903-11-07

**Authors:** J. Lawrence-Hamilton


### EDITOR'S LETTER-BOX.

[Our Correspondents are reminded that prolixity is a great bar to publication, and that brevity of style and conciseness of statement greatly facilitate early insertion.]

### FOOD: COOKING AND CATERING.

Mr. J. Lawrence-Hamilton, M.R.C.S., writes:—Taking into consideration the inferior digestive and nutritive values as well as the quality and condition of the food—especially the fresh food—of the poorer classes, it is a well-known fact that they pay comparatively more for it than their richer neighbours. In fact, the more or less spoilt sweepings of the fresh food markets are usually considered good enough for the poor, whose means do not allow them to go far afield for the daily diet.

As the chief expenditure of the poor is in fresh food, this question of catering presses directly upon their health, comfort, convenience, and wage-earning powers.

English manufacturers are too often unable to compete with their foreign rivals—more especially the Germans—because food is much cheaper and comparatively more nutritive in Germany than in England. The taxed German bread is actually, weight for weight, dearer than English bread, but it is much superior in digestive and life-sustaining powers, and so in the end practically the cheaper of the two.

Except along the German coast districts, fish is both dearer and worse than in England. Indeed, as a rule, in Germany fish is reserved exclusively as a diet for the well-to-do classes. Before fish reaches the great inland districts of Germany, and is sufficiently cheap for the poor and peasant classes to buy, it has reached such a stage of decomposition that they wisely avoid its purchase! This in a great measure accounts for the German poor and peasants' expressed dislike of fish.

Again, the average Englishwoman (in comparison with the German, French, Swiss, Belgian, or Italian) is a bad cook and a worse caterer. It is a common saying—even if somewhat exaggerated—that an ordinary Englishwoman wastes in catering what she does not spoil in cooking.

Cooking and catering should be amongst the compulsory subjects of every woman's education in the British Empire.

A Departmental Inquiry or a Royal Commission should immediately take up this subject of Imperial Domestic Cooking and Catering in a comprehensive practical spirit, and by sufficiently extensive inquiries impress on the leaders in politics and the press of the Empire the necessity of rescuing our imperial nation from further avoidable dangers and disasters to health, trade, and prosperity.

